# MULTIDIMENSIONAL AND INTERSECTIONAL APPROACHES TO ACCESSIBLE AND HEALTHY AGING IN DIVERSE POPULATIONS

**DOI:** 10.1093/geroni/igad104.0844

**Published:** 2023-12-21

**Authors:** Catherine Garcia

## Abstract

Aging is a complex process that affects different individuals in different ways. To ensure that healthy aging is accessible for all individuals, it is crucial to consider the unique needs of diverse populations through multidimensional and intersectional approaches. Multidimensional approaches recognize that aging is a complex process and that many different factors shape how individuals experience it. Intersectional approaches to aging consider individuals’ multiple identities and social locations, creating unique experiences and needs around aging. When these two approaches are combined, it is possible to create accessible and healthy aging for individuals from all backgrounds. The five presentations in this symposium center on two large and diverse racial and ethnic groups in the U.S. – Vietnamese and Puerto Rican older adults – groups underrepresented in studies of aging. Using data from the Vietnamese Aging and Care Survey (VACS), Miyawaki and colleagues examine the association between disability, chronic disease, and depression. The next set of presentations uses longitudinal data from the Puerto Rican Elderly Health Conditions Project (PREHCO). Quashie and colleagues explore whether neighborhood socioeconomic status moderates the relationship between living arrangements and cardiometabolic disease. Thompson and colleagues examine the relationship between hurricane stressors and cognition. Guinn and colleagues examine whether social support mediates the relationship between hurricane-related stressors and depression. Ballard and colleagues examine how hurricane-related stressors influence perceived stress. Overall, the results from these investigations show a need for authentic community and stakeholder engagement to develop, implement, and create sustainable interventions to promote healthy aging.

